# The Glycine Receptor Allosteric Ligands Library (GRALL)

**DOI:** 10.1093/bioinformatics/btaa170

**Published:** 2020-03-12

**Authors:** Adrien H Cerdan, Marion Sisquellas, Gilberto Pereira, Diego E Barreto Gomes, Jean-Pierre Changeux, Marco Cecchini

**Affiliations:** b1 Institut de Chimie de Strasbourg, UMR7177, CNRS, Université de Strasbourg, F-67083 Strasbourg Cedex, France; b2 Channel-Receptors Unit, Institut Pasteur, 75015 Paris, France; b3 Diretoria de Metrologia Aplicada às Ciências da Vida-Instituto Nacional de Metrologia, Qualidade e Tecnologia, Duque de Caxias 25.250-020, Brazil; b4 CNRS, URA 2182, F-75015, Collège de France, F-75005 Paris, France; b5 Kavli Institute for Brain & Mind, University of California San Diego, La Jolla, CA 92093, USA

## Abstract

**Motivation:**

Glycine receptors (GlyRs) mediate fast inhibitory neurotransmission in the brain and have been recognized as key pharmacological targets for pain. A large number of chemically diverse compounds that are able to modulate GlyR function both positively and negatively have been reported, which provides useful information for the development of pharmacological strategies and models for the allosteric modulation of these ion channels.

**Results:**

Based on existing literature, we have collected 218 unique chemical entities with documented modulatory activities at homomeric GlyR-α1 and -α3 and built a database named GRALL. This collection includes agonists, antagonists, positive and negative allosteric modulators and a number of experimentally inactive compounds. Most importantly, for a large fraction of them a structural annotation based on their putative binding site on the receptor is provided. This type of annotation, which is currently missing in other drug banks, along with the availability of cooperativity factors from radioligand displacement experiments are expected to improve the predictivity of *in silico* methodologies for allosteric drug discovery and boost the development of conformation-based pharmacological approaches.

**Availability and implementation:**

The GRALL library is distributed as a web-accessible database at the following link: https://ifm.chimie.unistra.fr/grall. For each molecular entry, it provides information on the chemical structure, the ligand-binding site, the direction of modulation, the potency, the 3D molecular structure and quantum-mechanical charges as determined by our *in-house* pipeline.

**Contact:**

mcecchini@unistra.fr

**Supplementary information:**

Supplementary data are available at *Bioinformatics* online.

## 1 Introduction

Glycine receptors (GlyRs) are pentameric ligand-gated ion channels (pLGICs) that mediate fast inhibitory neurotransmission in the spinal cord, brainstem and the retina (Lynch, 2004). Glycinergic inhibition is critical in many physiological processes from muscle regulation to essential sensory functions such as vision and audition (Lynch, 2009) and GlyR malfunction has been linked to neurological disorders in humans including hyperekplexia (Dutertre *et al.*, 2012), temporal lobe epilepsy and chronic inflammatory pain (Lynch *et al.*, 2017).

At the structural level, GlyR is by far the best-characterized pLGIC since several high-resolution structures in complex with modulatory ligands and in different conformations have been recently deposited. In a seminal work by Gouaux and coworkers, the structure of GlyR-α1 from *zebrafish* was solved in complex with the endogenous agonist glycine, the competitive agonist strychnine, or the allosteric agonist ivermectin (Du *et al.*, 2015). Although the physiological significance of the glycine-bound structure is still debated (Cerdan *et al.*, 2018; Dämgen and Biggin, 2020), these cryo-EM results provided atomistic representations of the orthosteric neurotransmitter site and one allosteric site located in the transmembrane domain. Subsequent X-ray crystallography of the *human* GlyR-α3 provided the details of glycine binding (Huang *et al.*, 2017a) and unveiled the existence of a novel modulatory site near the top of the extracellular domain (ECD) (Huang *et al.*, 2017b). Most recently, cryo-EM results described the binding mode of the negative modulator picrotoxin within the ion pore of GlyR (Kumar *et al.*, 2019) which is similar to the one observed in the γ-aminobutyric acid class A receptor (GABA_A_R) (Masiulis *et al.*, 2019). In addition to that, structural biology in homologous pLGICs highlighted the topographical location of several other regulatory sites, which are likely to exist in GlyR. For instance, X-ray crystallography of GABA_A_R chimeras demonstrated the existence of two neurosteroid-binding sites in the transmembrane domain of anionic pLGICs, one for potentiation and one for inhibition (Laverty *et al.*, 2017), whose relevance has been corroborated by subsequent X-ray crystallography of other GABA_A_R constructs solved in complex with pregnanolone (Miller *et al.*, 2017) and alphaxalone (Chen *et al.*, 2018). Although the relevance of such neurosteroid-binding sites has not been demonstrated yet in GlyR, modeling studies support the conclusion that the potentiation site in GABA_A_R is conserved in GlyR (Alvarez and Pecci, 2019). Similarly, X-ray crystallography of mutants of the proton-gated ion-channel GLIC allowed the identification of an inter-subunit site for potentiation by ethanol (Sauguet *et al.*, 2013) and propofol (Fourati *et al.*, 2018), whose existence in GlyR is corroborated by site-directed mutagenesis (Ahrens *et al.*, 2008; Lynagh and Laube, 2014). Last, X-ray crystallography of the 5-HT_3_ receptor in complex with granisetron (Basak *et al.*, 2019) and tropisetron (Polovinkin *et al.*, 2018) shed light onto the mechanism of negative modulation by tropeines. The existence of a tropeine-binding site for inhibition in GlyR is supported by a series of mutations at the lower rim of the orthosteric site that were shown to alter tropeine binding (Maksay *et al.*, 2009; Yang *et al.*, 2007). Overall, recent structural biology in GlyR and homologous pLGICs has provided the details of eight topographically distinct ligand-binding sites that are relevant for the allosteric modulation of GlyR.

A wide panel of small-molecule compounds has long been known to regulate the function of GlyR (Lynch *et al.*, 2017; Zeilhofer *et al.*, 2018). GlyR channels are activated by the homologous amino acids glycine, taurine and β-alanine (Mori *et al.*, 2002) and efficiently inhibited by the competitive antagonist strychnine (Brams *et al.*, 2011). In addition, a large number of positive (PAM) and negative allosteric modulators (NAM) based on unrelated chemical structures are known, which include cannabinoids, neurosteroids, tropeines, avermectines, general anesthetics, metal ions and toxins (Lynch *et al.*, 2017). Despite abundant pharmacological data have been collected on GlyR, little is known about the molecular mechanism(s) of modulation and even the ligand-binding site for most positive and negative modulators is unknown. In addition, non-linear effects such partial agonism or the bidirectional modulation by compounds like tropeines or the metal ions, which results in GlyR potentiation at sub-micromolar concentrations and inhibition at significantly higher concentrations (Yevenes and Zeilhofer, 2011), can be explained by non-exclusive ligand binding (Rubin and Changeux, 1966).

Based on existing literature, we provide here a curated database of small-molecule modulators with documented activity at homomeric GlyR-α1 and -α3. This library named GRALL, i.e. the glycine receptor allosteric ligands library, includes 218 unique chemical structures shipped with 3D molecular geometries and quantum-mechanical charges, which are ready for use in *in* *silico* pharmacology studies. Most importantly, the GRALL compounds are annotated according to the topographical location of their putative-binding site on GlyR using a five-level of confidence assignment. This structural annotation, which is unique among currently available drug databases, is expected to improve the performance of both ligand- and structure-based *in* *silico* approaches for drug discovery. The GRALL library provides the most complete collection of chemical structures for the allosteric regulation of a pharmacologically relevant neurotransmitter receptor.

## 2 Glycine Receptor Allosteric Ligands Library

**Fig. 1.**
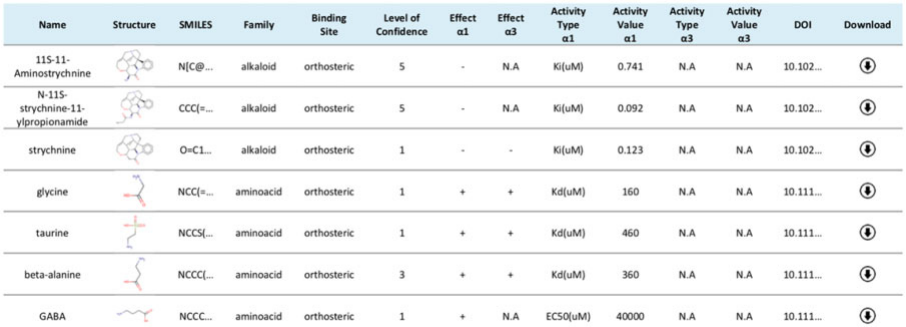
Screenshot from the GRALL website (https://ifm.chimie.unistra.fr/grall). GRALL can be downloaded as a combination of CSV (data) and multi-MOL2 (3D structures) files or accessed online. All compounds are displayed as rows in a table. For each compound, the chemical name, the 2D chemical structure, the isomeric SMILES, the chemical family, the binding site (if known and the level of confidence), the type of activity measurement, the potency against GlyR-α1 and/or -α3, the direction of modulation (i.e. PAM, NAM or absence of modulation) on GlyR-α1 and/or -α3, the DOI of the original publication reporting the functional assays and a link to the 3D structure of the compound in MOL2 format is available. A text-based search engine permits to filter the chemical library. Each column of the database can be directly sorted. Not Available (N.A) is used when the information was not found in the literature

Based on the existing literature (Lynch *et al.*, 2017; Zeilhofer *et al.*, 2018 and references therein) and more recent publications, a collection of 218 unique small molecules with documented modulatory activity on GlyR-α1 and -α3 has been compiled. This collection named GRALL contains several agonists and antagonists, a large number of positive and negative allosteric modulators and a significant fraction of experimentally inactive compounds. GRALL is distributed as a web-accessible database at the following link; https://ifm.chimie.unistra.fr/grall. For each entry, the database allows for immediate visualization of the 2D chemical structure of the compound and provides information on its ligand-binding site, the direction of modulation (positive or negative), the potency and the 3D molecular structure and charge distribution in the most likely protonation and tautomeric state (see Fig. 1). The hyperlink at each entry provides access to the 3D representation in MOL2 format, which is suitable for straightforward visualization via popular software like Avogadro (Hanwell *et al.*, 2012), PyMOL (Schrödinger, LLC, 2015), VMD (Humphrey *et al.*, 1996) or UCSF Chimera (Pettersen *et al.*, 2004). The molecular topology, 3D coordinates and partial atomic charges based on quantum-mechanical calculations were determined using the protocol described in the Section 4.

**Fig. 2.**
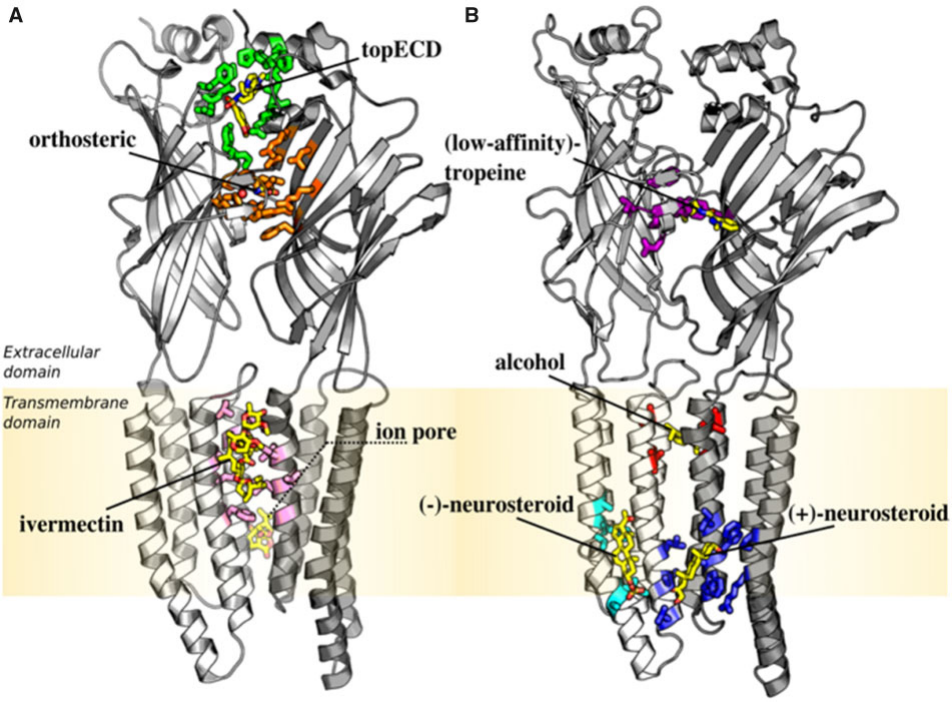
Topographical location of the actual/putative regulatory sites at GlyR by structural biology. (**A**) Regulatory sites illuminated by high-resolution structures of GlyR. From top to bottom: (i) the allosteric inter-subunit extracellular site (top-ECD) that accommodates the positive allosteric modulator AM-3607 is shown in green; (ii) the orthosteric site that binds the endogenous neurotransmitter glycine is in orange; (iii) the allosteric inter-subunit site in the transmembrane domain that binds the positive allosteric modulator ivermectin (ivermectin site) is in pink; (iv) the allosteric site for negative modulation by picrotoxin (ion pore site) is in the background. Coordinates for glycine, the allosteric modulators AM-3607 and ivermectin were extracted from the X-ray structure of GlyR-α3 (PDB: 5VDH; Huang *et al.*, 2017a). Coordinates for picrotoxin were extracted from the X-ray structure of GABA_A_R-α1β3γ2 (PDB: 6HUG; Masiulis *et al*., 2019); they are consistent with the most-recent cryo-EM results in GlyR-α1 (Kumar *et al*., 2019). (**B**) Putative regulatory sites illuminated by high-resolution structures of homologous pLGICs with concordant evidence at GlyR. From top to bottom: (i) the extracellular site for the low-affinity tropeine-binding site is shown in purple with granisetron bound; (ii) the inter-subunit transmembrane site for alcohol binding is in red with propofol bound; (iii) the allosteric intra-subunit binding site for inhibition by neurosteroids or (−)-neurosteroid site is shown in cyan with pregnenolone sulfate (PS) bound; (iv) the allosteric inter-subunit binding site for potentiation by neurosteroids or (+)-neurosteroid site is shown in dark blue with tetrahydrodeoxycorticosterone (THDOC) bound. Coordinates for THDOC and PS were extracted from the X-ray structures of GLIC-GABA_A_R-α1 chimera (PDB: 5OSB) and (PDB: 5OSC) (Laverty *et al*., 2017), respectively, after structural alignment to GlyR-α3 in PyMOL (Schrödinger, LLC, 2015). Coordinates for granisetron were extracted from the high-resolution structure of 5-HT_3_A receptor (PDB: 6NP0; Basak *et al*., 2019). Coordinates for propofol were extracted from the high-resolution structure of GLIC F238A/N239A (PDB: 5MVM; Fourati *et al*., 2018). In all cases, residues that are known to be involved in binding of allosteric modulators at GlyR are color-coded

In this version, GRALL contains 122 potentiators, 53 inhibitors and 34 inactive compounds at GlyR-α1, which correspond to 55%, 24% and 16% of the entire collection, respectively (see Supplementary Table S1). Similar numbers hold for GlyR-α3. Since the inactive compounds are chemically related to the known actives, i.e. they belong to the same chemical series but display no activity in functional assays; they provide an excellent benchmark for the development of predictive models of allosteric modulation at GlyR. Most importantly, about one-half of the GRALL compounds are annotated within one of the eight regulatory sites highlighted by structural biology using a five-level of confidence assignment (see Table 1 and Fig. 2). These levels of annotations (from most reliable to least reliable) are based on: high-resolution structures of GlyR (Level 1); high-resolution structures of homologous pLGICs with concordant evidence in GlyR (Level 2), functional studies in conjunction with site-directed mutagenesis (Level 3); modeling data (Level 4); or chemical similarity to annotated compounds (Level 5); see Section 4 for details. The structural annotation in GRALL highlights the existence of topographically distinct ligand-binding sites on GlyR with distinct modulatory properties (see Table 1): three of them for positive modulation (i.e. the ivermectin, the top-ECD and the alcohol-binding sites); three for negative modulation (i.e. the ion pore, the low-affinity tropeine and the (−)-neurosteroid-binding sites); and two (i.e. the orthosteric and the (+)-neurosteroid-binding sites) that can be used for both activation and inhibition. This perhaps surprising result can be understood considering that most modulatory sites are located at the subunit–subunit interface, which reshape significantly during the functional isomerization of the ion channel (Cecchini and Changeux, 2015). Therefore, a complete structural annotation of the modulatory ligands in GlyR would require not only the specification of the ligand-binding site, but also an annotation of the conformational state of the receptor which the compound has the highest affinity to; e.g. glycine and strychnine are both orthosteric ligands but the former is an agonist and binds preferentially to the active state, whereas the latter is an antagonist and binds preferentially to the resting state.

**Table 1.** Structurally annotated modulators in GRALL

Site	Total	PAM	NAM
Orthosteric	7	4	3
Ivermectin	7	7	/
Top-ECD	29	29	/
Ion pore	21	/	21
(Low-affinity)-tropeine	11	/	11
Alcohol	8	8	/
(+)-Neurosteroid	10	8	2
(−)-Neurosteroid	4	/	4
Total	97	56	41

The structurally annotated fraction of GRALL provides an unprecedentedly curated chemical database for the allosteric modulation of an important pharmacological target, which can be most effectively explored by chemoinformatics or machine-learning approaches (Lo *et al.*, 2018; Xu and Hagler, 2002). In fact, information on the binding site will remove ambiguities between ligands with similar modulatory activity but targeting topographically distinct sites (see Supplementary Figs S1–S8) or different conformational states of the receptor, which is expected to improve the predictivity of ligand-based drug-discovery approaches. We note that this information is currently missing in general databases such as DrugBank (Wishart *et al.*, 2018), ChEMBL (Gaulton *et al.*, 2012) or ZINC (Sterling and Irwin, 2015) and even in more specialized ones like ASD (Huang *et al.*, 2011).

Besides the structurally annotated compounds, 91 chemical entities with reported modulatory activity in GRALL lack of structural information. These small-molecule modulators, which include 76 PAMs and 15 NAMs, can be grouped in seven chemical families: potentiating tropeines (24), cannabinoids (11), glutamate analogs (9), phenylalanine derivatives (8), general anesthetics (5), ginkgolic-acid (1), gelsemine (1) and 32 chemically diverse compounds recently discovered by high-throughput screening (HTS) (see Supplementary Table S2). The classification by family not only highlights a certain degree of chemical similarity among the modulatory ligands but also suggests that compounds within a family are likely to target the same binding site. Although this information is not useful for structure-based drug discovery because the definition of the binding site is missing, it provides valuable information for ligand-based screening campaigns.

More than 80% of the GRALL compounds are provided with potency measurements. For one quarter of them, potencies are given in the form of dissociation or inhibition constants (*K*_d_ or *K*_i_), whereas the rest as half-maximal effective concentrations, i.e. EC50 or IC50. Although measured EC50 or IC50 values depend on the actual concentration of neurotransmitter in the assay, which would prevent a direct comparison of results collected from different publications, these data are still amenable to a coarse classification. By grouping ligands into four classes (see Supplementary Table S3), i.e. highly potent (<100 nM), potent (100 nM to 1 μM), intermediate (1–10 μM) and weak (>10 μM), the GRALL collection indicates that: (i) the majority of compounds is active in the micromolar range; (ii) most compounds (∼66%) are PAMs; (iii) a large number of potent or highly potent modulators exists (53) but only 12 among them are NAMs; (iv) a small fraction of compounds (12), which are mostly endocannabinoids, displays opposite modulatory effects at GlyR-α1 or -α3. Moreover, groups of congeneric ligands for which consistent IC50 or EC50 values are available, e.g. data collected by the same research group and under comparable experimental conditions, provide useful benchmarks for the development of predictive models per modulatory site.

Last, the allosteric modulation of GlyR by a certain number of compounds has been analyzed by radioligand displacement experiments using a ternary allosteric model for the bi-cooperative modulation (Maksay and Bíró, 2002). Application of such a model, which neglects desensitization, provided estimates of the ligand-binding constants at both resting (apo) and active (glycine-bound) states, whose ratio β quantifies the cooperativity of allosteric ligand binding with the endogenous neurotransmitter. Interestingly, the value of β, which was shown to discriminate between PAMs and NAMs (Maksay and Bíró, 2002), provides quantitative estimates of the difference in the ligand-binding affinity for active versus resting; i.e. $\Delta \Delta G_{b}=\Delta G_{b}\left(A\right)-\Delta G_{b}\left(R\right)=RT\ln\beta$. Provided that high-resolution structures of the receptor are available in the active and resting forms, which is the case for GlyR, and that the ligand-binding site is structurally known, as provided by GRALL, cooperativity factors (β) can be accessed independently by binding free energy calculations (Cournia *et al.*, 2017). As such, the subset of GRALL compounds for which cooperativity factors are available provides a unique benchmark for the development of predictive strategies for the rational design of positive and negative allosteric modulators by state-based or conformation-based pharmacology. More generally, the increasing availability of high-resolution structures of GlyR in complex with ligands in combination with molecular simulations opens to quantitative analyses of non-exclusive ligand binding, which can be used as a theoretical framework to rationalize partial agonism, i.e. non-exclusive binding to distinct conformational states of the receptor (Yu *et al.*, 2019), or the bidirectional modulation of compounds like tropeines, i.e. non-exclusive binding to distinct binding sites (Maksay *et al.*, 2009). This concept is reminiscent of ligand efficacy versus efficiency (Nayak and Auerbach, 2017; Nayak *et al.*, 2019).

## 3 Conclusions

We have reported on the GRALL database, which is a carefully curated, web-accessible library of small-molecule modulators of GlyRs. The library contains 218 unique chemical entities including agonists, antagonists, positive and negative allosteric modulators with documented activity and potency, which are ready for use in *in* *silico* drug-discovery projects. By making use of an original annotation based on structural data at high-resolution, site-directed mutagenesis and modeling, GRALL provides the actual/putative topographical location of the ligand-binding site for a large fraction of compounds. The adopted procedure extends the number of annotated compounds from 8 (based on high-resolution structures of GlyR) to 97, whose binding sites have been assigned using a level of confidence from 1 to 5. The classification of compounds based on their putative binding site highlights the extreme chemical diversity of GlyR modulators that bind to distinct regulatory sites, which needs to be accounted for in drug-discovery campaigns targeting GlyR or analogous channels. Therefore, the structural annotation along with the availability of experimentally inactive compounds in GRALL is expected to facilitate the development of predictive models for allosteric drug design in neurotransmitter receptors.

Straightforward statistics on GRALL highlights: (i) the lack of structural information concerning pharmacologically relevant classes of GlyR modulators such as tropeines (i.e. the high-affinity site) and cannabinoids; (ii) a striking paucity of potent NAMs targeting GlyR; and (iii) the lack of cooperativity factors e.g. extracted from radioligand displacement experiments for the majority of known modulators. These observations along with the intriguing finding that the (+)-neurosteroid site is the only allosteric site that is so far able to accommodate both PAMs and NAMs, are genuine results from GRALL, which provide clear directions for future research.

Overall, the manually curated information in GRALL, i.e. the annotation of the binding site, the modulatory activity, the 3D structure and quantum charges per molecular entry, will be instrumental for the development of predictive pharmacological approaches for the allosteric modulation of GlyR, pLGICs and allosteric proteins more generally.

## 4 Materials and methods

### 4.1 Data collection

Small-molecule compounds with reported modulatory activity on GlyR-α1 or -α3 were collected from the existing literature. The 2D molecular structures were extracted from the original publications when this information was available or drawn with Marvin (Chemaxon, 2019). All defined stereo centers were encoded in the structure. If the compounds were tested experimentally as a racemic mixture, or the stereochemistry was not specified, the corresponding centers were labeled, and all stereoisomers enumerated. Concerning the modulatory activity reported in GRALL, we favored measurements of *K*_d_/*K*_i_ over EC50/IC50 over mean/maximum potentiation. When multiples publications were found that report on the activity of one given compound, the value in the publication that included the largest number of activity measurements was kept for reasons of consistency; this choice allows for a fair comparison and ranking of congeneric compounds. Standardization into canonical SMILES (Simplified Molecular Input Line Entry Specification) was achieved using RDKit (Landrum, 2006). The resulting SMILES were then used for ligand preparation by our *in-house* pipeline; see below. For the generation of the 2D images displayed on the website, the KNIME software (Berthold *et al.*, 2007) was used.

### 4.2 Ligands preparation

Starting with isomeric, canonical SMILES, our pipeline automatically prepares 3D molecular structures accounting for the stereochemistry, the tautomerization state, the protonation state, geometry optimization and charge determination at the quantum level of theory. This pipeline is coded in Python3 (van Rossum and Drake, 1995) and interfaces popular software like RDKit, Marvin, Antechamber and Gaussian for specific manipulations. A short description of the preparation steps follows.

#### 4.2.1 Tautomeric state

The dominant tautomeric state of the ligand was determined using *Marvin cxcalc* (ChemAxon, 2019). For this purpose, different protonation states of the ligand at pH 7.0 were considered. All tautomers and protonation isomers with a probability >10% were retained and enumerated as ‘LIGAND_X’, with X being the number of the isomer/protonation state. Last, explicit hydrogens were added. The following parameters were used for the *cxcalc* execution: pathlength (default: 4), protect aromaticity (default: true), protect charge (default: true), exclude antiaromatic compounds (default: true), protect ester groups (default: true).

#### 4.2.2 Enantiomers

Using *cxcalc stereoisomers* of Marvin (ChemAxon, 2019), all ligand enantiomers were enumerated while protecting the stereo centers (i.e. asymmetric carbons and double stereo bonds) that were explicitly defined. Generated enantiomers were uniquely identified as ‘LIGAND_X_Y’, with Y being the number of the enantiomer generated.

#### 4.2.3 Conformers

For each ‘LIGAND_X_Y’ entry, the conformer of lowest energy was determined using Marvin *cxcalc conformers* (ChemAxon, 2019) and the Merck Molecular Force Field (MMFF94) (Halgren, 1996). Parameters used: diversity limit (default: 0.1), two conformers having a root-mean-square deviation in Angstrom lower than the diversity limit were considered the same and discarded; optimization (default: 1 = normal).

#### 4.2.4 Partial charges

Partial atomic charges were computed using the RESP (Restrained ElectroStatic Potential) protocol (Bayly *et al.*, 1993) as implemented in Antechamber17.3 (Wang *et al.*, 2001). Computation of RESP charges requires knowledge of the electrostatic potential as determined by *ab initio* calculations. These calculations were performed using Gaussian09 (Frisch *et al.*, 2009). Using the input file created by Antechamber, Gaussian09 optimizes the geometry of the ligand at the Hartree-Fock level of theory and an electrostatic potential grid around the ligand is generated. Post-processing of the grid by Antechamber produces RESP quantum-mechanical charges. All parameters in Antechamber were set as default using general AMBER force field (GAFF) atom typing.

### 4.3 Structural annotation

The high-resolution structures of GlyR and other pLGICs in complex with agonists, antagonists, PAMs and NAMs that are known to affect GlyR function provide 3D representations of eight topographically distinct regulatory sites in GlyR with atomic resolution (Fig. 2). Starting from this evidence, the structural annotation provided in GRALL was assigned as follows. Compounds solved in complex with GlyR by X-ray or cryo-EM were annotated with a level of confidence 1. Compounds solved in complex with other pLGICs by X-ray or cryo-EM in a regulatory site not displayed at GlyR but with concordant evidence in GlyR (see Supplementary Material) were annotated with a level of confidence 2. Compounds for which site-directed mutagenesis at one actual/putative regulatory site was shown to affect the allosteric modulation in functional assays were annotated with a level of confidence 3. Compounds for which modeling or computational studies support binding one actual/putative regulatory site were annotated with a level of confidence 4. Last, compounds that are chemically similar to previously annotated compounds as measured by an average pair-wise distance between strings of chemical descriptors (see below) were annotated to the same binding site with a level of confidence 5. Note that compounds for which site-directed mutagenesis data fall outside the boundaries of the regulatory sites characterized by structural biology, e.g. the cannabinoids, were not annotated. The latter avoids over-interpretations of sparse mutagenesis data, which may include the effect of allosteric mutations.

### 4.4 Chemical similarity

Congeneric compounds with high-structural similarity or consistent Structure-Activity Relationship (SAR) with one lead compound annotated at Level 1, 2, 3, or 4 have not been necessarily explored by structural biology or site-directed mutagenesis, although they are likely to bind to the same regulatory site. In order to provide an objective annotation for these compounds the Morgan/Circular fingerprints as implemented in RDKit (Extended Connectivity FingerPrints-like; Rogers and Hahn, 2010) were used with 1024 bits and a radius of 2 to quantify chemical similarity. By analyzing the similarity of the GRALL compounds with the fraction of ligands annotated at Level 1 or 2 (structural biology), we found that a Tanimoto coefficient (T_c_) of 0.4 used as a distance threshold between Morgan fingerprints is appropriate to group congeneric compounds that exert comparable modulation at GlyR. Therefore, compounds with a maximal pair-wise T_c_ > 0.4 relative to ligands in Level 1 to 4 were annotated to the same binding site with a level of confidence 5. We note that this protocol produced no unexpected annotation, while rationalizing the assumption of similarity made by the authors in the various publications. One peculiarity concerns the neurosteroid compounds, for which the threshold T_c_ > 0.4 with Morgan fingerprints was not able to discriminate between (+)- and (−)-neurosteroid-site binders. Since it was found that negatively charged neurosteroids tend to act as negative modulators (Maksay *et al.*, 2001) and (Laverty *et al.*, 2017), these compounds are expected to bind the (−)-neurosteroid site. Indeed, this structure-activity relationship is consistent with the presence (in GABA_A_R and GlyR) of positively charged residues in the lower part of the (−)-neurosteroid site and their absence at the (+)-neurosteroid site. Therefore, in addition to the Tanimoto coefficient a filter based on the formal charge of the molecule was introduced to assign negatively charged ligands to the (−)-neurosteroids site and all others to the (+)-neurosteroid site with a level of confidence 5.

### 4.5 Future updates

GRALL aims at being a curated and up-to-date database of GlyR modulators. For this purpose, special care was taken to develop automated procedures to process new molecular entities and update the database. Because human processing of a constantly growing literature is challenging but critical to ensure the highest level of accuracy, we encourage researchers to contact us and submit missing information (e.g. new compounds, structures, activity measurements, etc.) via the preformatted form available on the website (https://ifm.chimie.unistra.fr/submission-ligand). Upon verification, the transmitted information will be added to the database and published online.

## Supplementary Material

btaa170_Supplementary_Data
