# Supplementary material for: The Glycine Receptor Allosteric Ligands Library (GRALL)
Source: Bioinformatics. 2020 Mar 12;36(11):3379–84. doi: 10.1093/bioinformatics/btaa170 (PMC7267813; doi:10.1093/bioinformatics/btaa170)
Supplement: btaa170_Supplementary_Data [file btaa170_supplementary_data.docx]

**Table S1.** Basic statistics of GRALL.

| Class | GlyR-α1 | GlyR-α3 |
| --- | --- | --- |
| PAM | 122 | 72 |
| NAM | 53 | 35 |
| Inactive | 34 | 28 |
| Total | 209 | 135 |

**Table S2.** Non-structurally annotated modulators in GRALL.

| Family | Total | PAM | NAM |
| --- | --- | --- | --- |
| Cannabinoids | 11 | 7 | 4 |
| Potentiating tropeines | 24 | 24 | / |
| GA | 5 | 5 | / |
| Glutamates | 9 | 7 | 2 |
| Gelsemine | 1 | 1 | / |
| Phenylalanine | 8 | 5 | 3 |
| Ginkgolic acid | 1 | 1 | / |
| HTS | 32 | 26 | 6 |
| Total | 91 | 76 | 15 |

**Table S3.** Ligand classification by potency.

| Class | Highly potent  (< 100 nM) | Potent  (100 nM to 1μM) | Intermediate  (1-10 μM) | Weak  (> 10 μM) | Total |
| --- | --- | --- | --- | --- | --- |
| PAM | 17 | 25 | 36 | 23 | 101 |
| NAM | 6 | 7 | 24 | 18 | 55 |
| Total | 23 | 32 | 60 | 41 | 156 |

**Structural definition of regulatory sites at GlyR**

List of residues forming the four regulatory sites at GlyR that have been characterized by X-ray crystallography or cryo-EM studies (Figure 2A):

*topECD site* (PDB: 5TIO):

(+)-subunit: I28, R29, F32, G160, Y161

(-)-subunit: P10, F13, L14, Y78, L83, D84, L85

*orthosteric site* (PDB: 5CFB):

(+)-subunit: F99, E157, F159,Y202, T204, F207

(-)-subunit: F44, F63, R65, L117, R119, L127, S129

*ivermectin site* (PDB: 5VDI):

(+)-subunit: S267, V280, A288, L291, F295

(-)-subunit: I225, I229, P230, L232, L233, I236

*ion pore site* (PDB: 6UD3):

A254 (2’), T258 (6’), T258 (6’), L261 (9’)

**Structural definition of putative regulatory sites at GlyR**

List of residues forming putative ligand-binding sites at GlyR that have been structurally characterized in other pLGICs and whose relevance in GlyR is supported by concordant pieces of evidence (Figure 2B):

*low-affinity tropeine site* (PDB: 6NPO):

(+)-subunit: N102, R199, K200, Y202, F207

(-)-subunit: F63

Justification :

5-HT_3_R co-crystallized with granisetron (PDB: 6NPO)

5-HT_3_R co-crystallized with tropisetron (PDB: 5HIS)

Tropeines are interesting compounds that display bimodal modulation at GlyR (Gábor Maksay and Bíró 2002). While some of them behave as PAMs at low concentration, the majority of them act as NAMs at higher concentrations. This peculiar behavior has been rationalized by proposing the existence of two distinct tropeine-binding sites, one for potentiation at low ligand concentration (i.e. the high-affinity site), and one for inhibition at higher ligand concentration (i.e. the low-affinity site). Site-directed mutagenesis highlighted two different clusters of residues abolishing selectively potentiation or inhibition by tropisetron and 3α-(3’-methoxybenzoyloxy)-nortropane (MBN) (Yang et al. 2007; Gábor Maksay et al. 2009). By displaying the structural location of residues that modulate tropeine inhibition at GlyR, i.e. those corresponding to the *low-affinity tropeine site,* it appears that they cluster in proximity to the *orthosteric site*, where both granisetron and tropisetron were co-crystallized at 5-HT_3_R. Based on this evidence, tropeine ligands that display GlyR inhibition at high ligand concentration were structurally annotated to the *low-affinity tropeine site* (Figure S7). By contrast, tropeines that act as PAMs at low ligand concentration were left at *NA*, i.e. no annotation, because no high-resolution structure describing their binding mode at GlyR or homologous pLGICs is currently available.

*alcohol site* (PDB: 5MVM):

(+)-subunit: A288

(-)-subunit: Q266 (14’), S267 (15’), S270 (18’)

Justification:

GLIC F238A/N239A co-crystallized with propofol (PDB: 5MVM)

GLIC F238A co-crystallized with ethanol (PDB: 4HFE)

Site-directed mutagenesis at positions 14’ and 15’ in GLIC (F238A/N239A) was recently shown to promote alcohol binding at an intersubunit transmembrane site by X-ray crystallography, thereby promoting potentiation by propofol and ethanol (Fourati et al. 2018). Although alcohols are known to potentiate anionic rather than cationic pLGICs, the observation in GLIC is consistent with this knowledge, as anionic channels display significantly smaller side chains than cationic at these positions in the transmembrane domain. Finally, mutagenesis studies have shown that the upper part of the transmembrane helices M2 and M3 (i.e. residues 14’, 15’, 18’, and A288) are directly implicated in GlyR potentiation by alcohols (Lynagh and Laube 2014), which correspond to the intersubunit cavity that accomodates propofol and ethanol in GLIC mutants. Based on this evidence, propofol derivatives and linear alcohols were annotated to the *alcohol site* (Figure S8).

*(-)-neurosteroid site* (PDB: 5OSC):

(+)-subunit: K321, I322, C329, F330, A333

Justification:

GABA_A_R co-crystallized with pregnenolone-sulfate (PDB: 5OSC)

Analysis of inhibitory neurosteroids at GlyR (G. Maksay, Laube, and Betz 2001) and the recent high-resolution structure of GABA_A_R in complex with pregnenolone sulfate (Laverty et al. 2017) indicate a strong structure-activity relationship (SAR) between the presence of a negative charge in the neurosteroid ligand and its negative modulation at GlyR. This SAR is consistent with the presence of positively charged residues (both in GABA_A_R and GlyR) in the lower part of the pregnenolone sulfate binding site, here referred to as *(-)-neurosteroid*. Based on this evidence, all negatively charged neurosteroid ligands were assigned to the *(-)-neurosteroid* *site* (Figure S4).

*(+)-neurosteroid site (*PDB: 5OSB*)*:

(+)-subunit: L299, A302, A303, V307

(-)-subunit: I236, W239, V240, W243, R327, P331

Justification:

GABA_A_R co-crystallized with THDOC (PDB: 5OSB)

GABA_A_R co-crystallized with pregnanolone (PDB: 5O8F)

GABA_A_R co-crystallized with alphaxalone (PDB: 6CDU)

Neurosteroids like pregnanolone, tetrahydrodeoxycorticosterone, and alphaxalone that act as allosteric modulators at GlyR have been recently co-crystallized in GABA_A_R (Miller et al. 2017) and (Laverty et al. 2017). These high-resolution structures provide consistent evidence that non-formally charged neurosteroids bind at the interface between subunits to a site, here referred to as the *(+)-neurosteroid site,* that is structurally distinct from the one for negatively charged neurosteroid binding. The relevance of this binding site at GlyR is supported by recent modeling studies showing that both pregnanolone and its stereoisomer allopregnanolone may stably bind the *(+)-neurosteroid site* in GlyR. Based on this evidence, all neurosteroid ligands with no negative charge were assigned to the *(+)-neurosteroid* *site* (Figure S3).

**Annotated modulatory ligands at GlyR**

The 2D chemical structure of the GRALL ligands provided with a structural annotation is presented below (see Figure S1-S8). Note how chemical similarity is high within a binding site (same table) but strikingly low among pairs of binding sites (different tables). For this reason, compounds that share the same function (i.e. PAM or NAM) but target different modulatory sites appear as chemically incomparable. Indeed, one aim of the GRALL classification is removing the ambiguity that exists among functionally equivalent modulators that target topographically distinct regulatory sites.

| 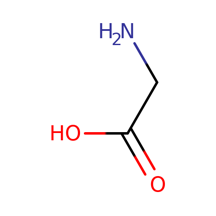 | 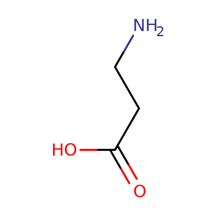 | 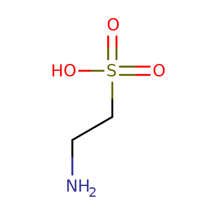 | 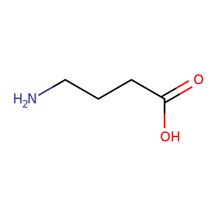 |
| --- | --- | --- | --- |
| 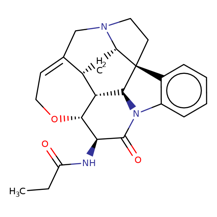 | 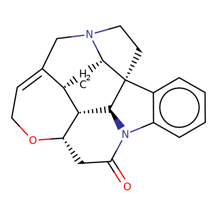 | 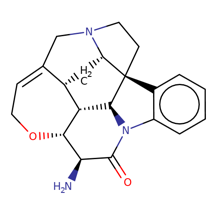 |  |

**Figure S1.** Chemical structures of GRALL ligands binding to the *orthosteric site*. The molecules squared by a red line are competitive antagonists, the others are agonists or partial agonists.

| 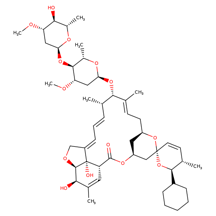 | 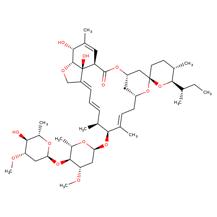 | 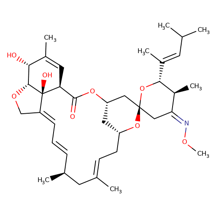 | 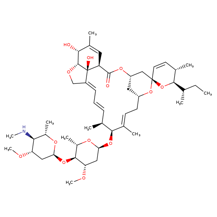 | 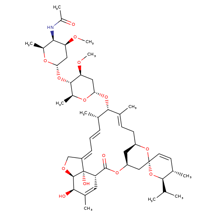 | 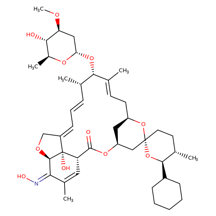 |
| --- | --- | --- | --- | --- | --- |
| 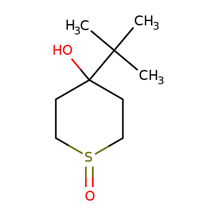 |  |  |  |  |  |

**Figure S2.** Chemical structures of GRALL ligands binding to the *ivermectin site*. All known ligands binding there are PAMs.

| 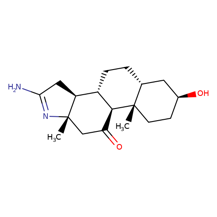 | 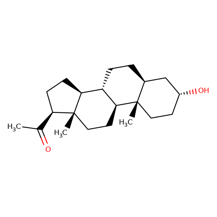 | 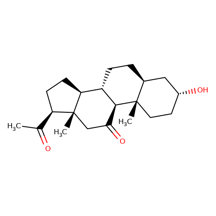 | 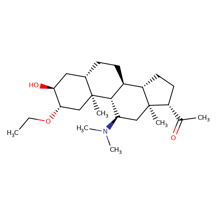 | 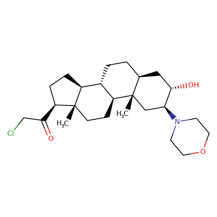 |
| --- | --- | --- | --- | --- |
| 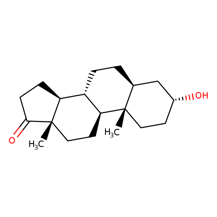 | 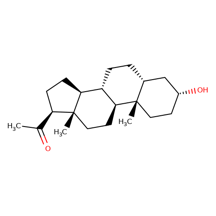 | 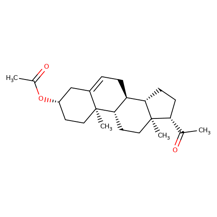 | 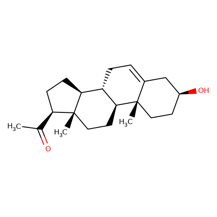 | 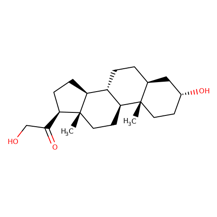 |

**Figure S3.** Chemical structures of GRALL ligands binding to the *(+)-neurosteroid site*. The two molecules squared by a red line are NAMs, while all others are PAMs. The chirality of the carbon conventionally numbered as the fifth one is the discriminating factor between PAM and NAM within the *(+)-neurosteroid site.*

Two neurosteroids were experimentally probed as NAM in GlyR, although they do not carry formal charges (i.e., pregnanolone and RU-135), contrary to the negatively charged steroids that bind to the *(-)-neurosteroid site*. Interestingly, while pregnanolone act as a NAM in GlyR, its stereoisomer allopregnanolone is a PAM (Weir et al. 2004), highlighting the importance of the chirality of the fifth carbon (5α vs. 5β). Recently it was shown by computational methods (Alvarez and Pecci 2019) that both stereoisomers can stably bind the same *(+)-neurosteroid site* in GlyR while proceeding to different binding modes.

To our knowledge, GRALL is the first report that permits to link the inhibitory effect of the steroid-derivative RU-5135 and its 5β configuration (i.e., similar to pregnanolone), by analyzing the subset of steroids that we annotated within the *(+)-neurosteroid site*.

| 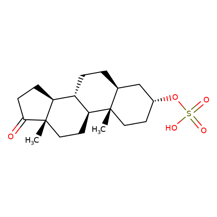 | 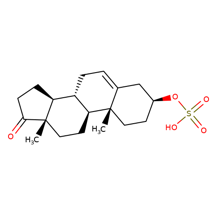 | 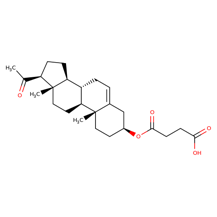 | 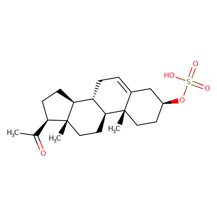 |
| --- | --- | --- | --- |

**Figure S4.** Chemical structures of GRALL ligands binding to the *(-)-neurosteroid site*. All known ligands binding there are NAMs.

| 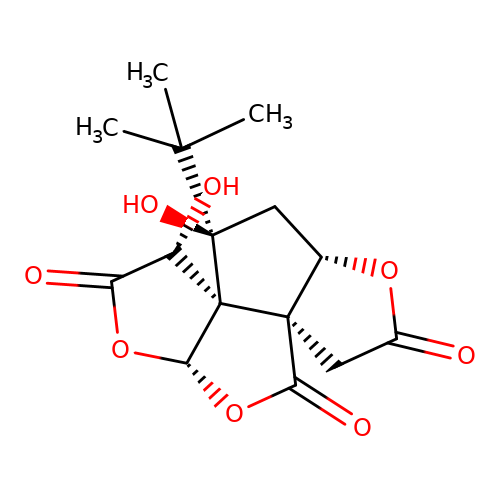 | 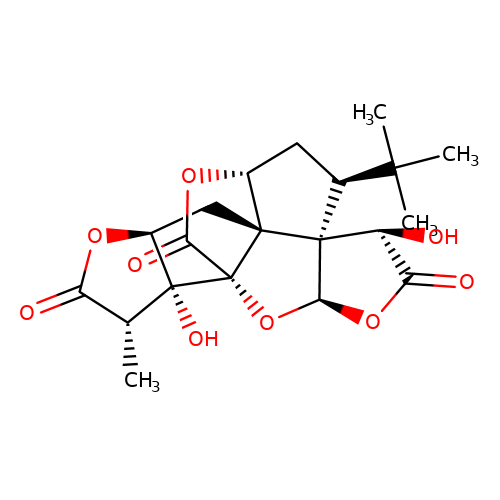 | 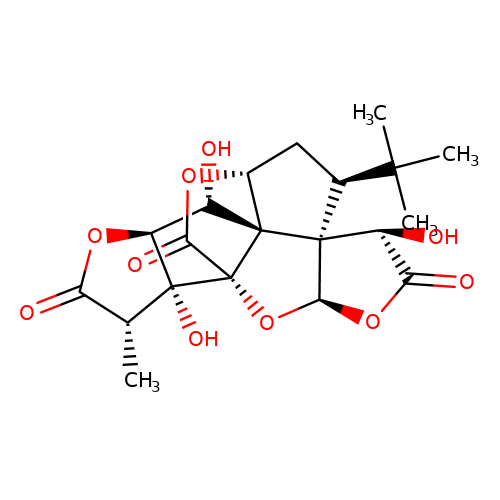 | 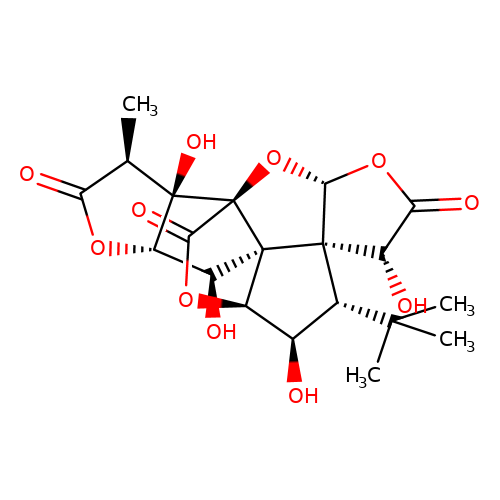 | 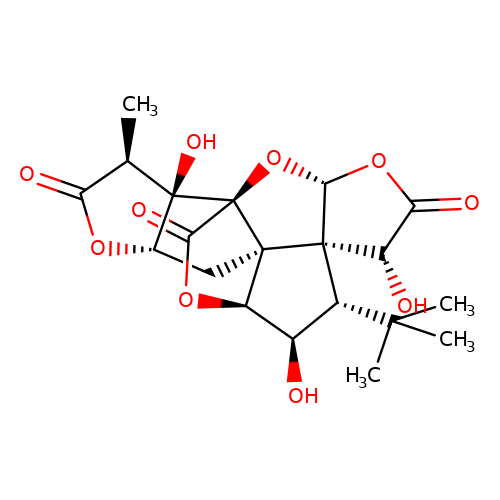 | 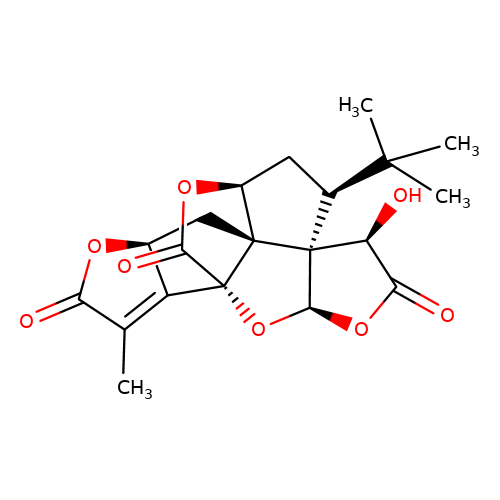 |
| --- | --- | --- | --- | --- | --- |
| 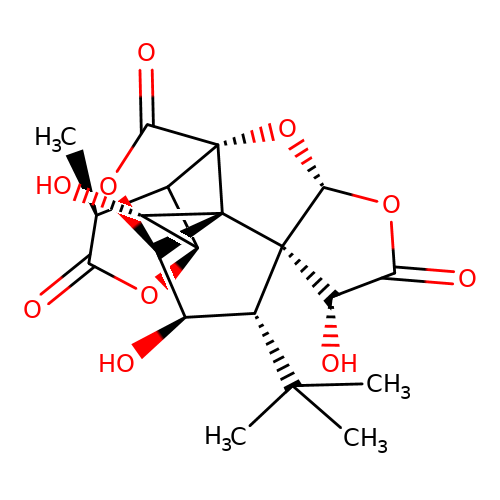 | 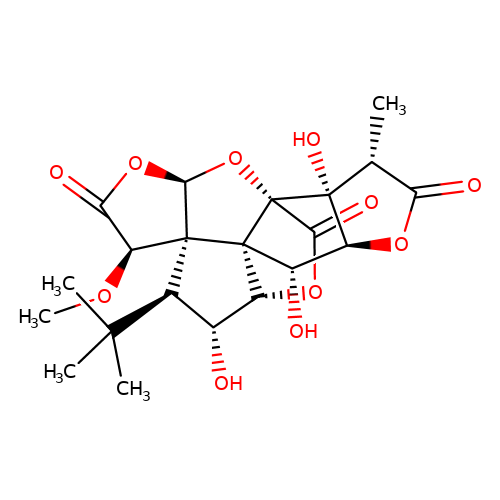 | 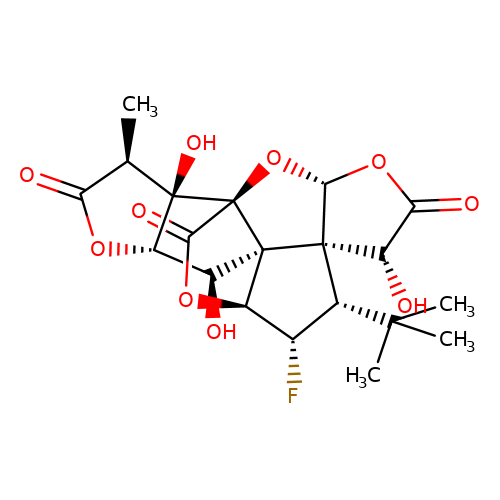 | 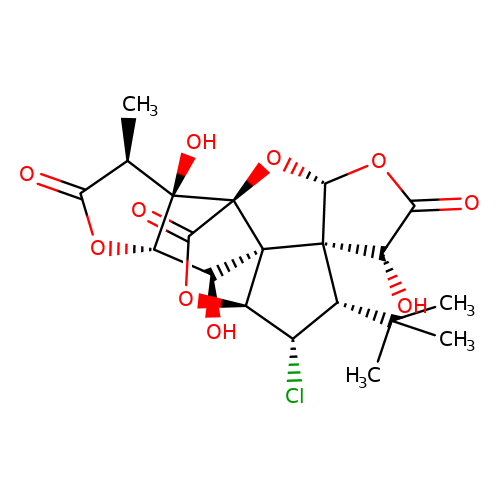 | 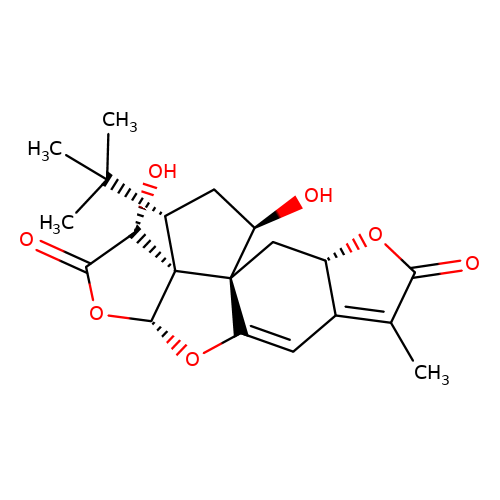 | 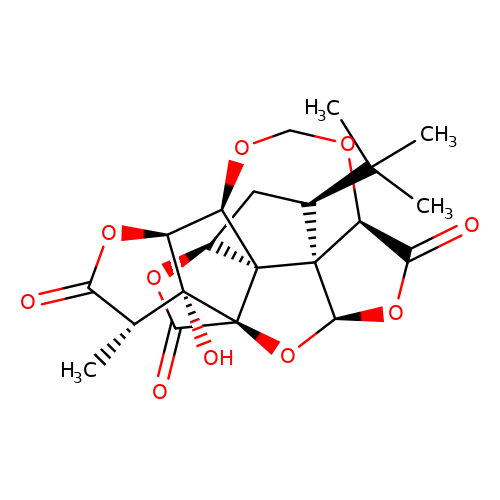 |
| 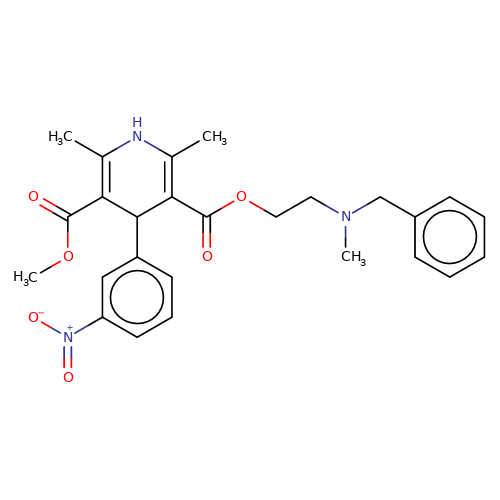 | 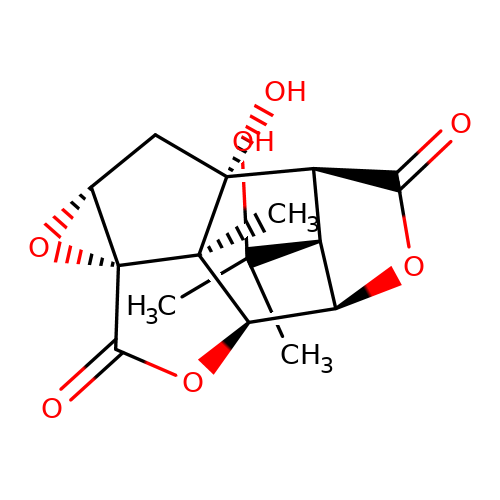 | 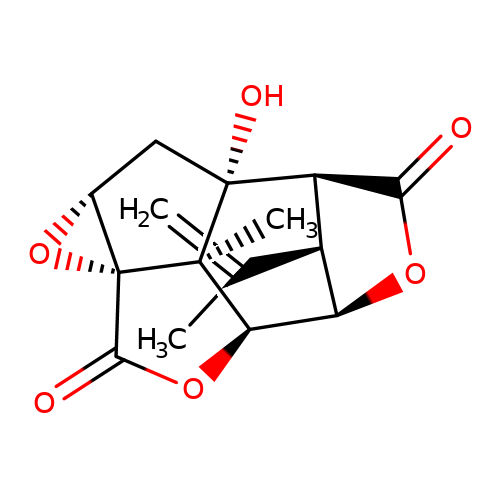 | 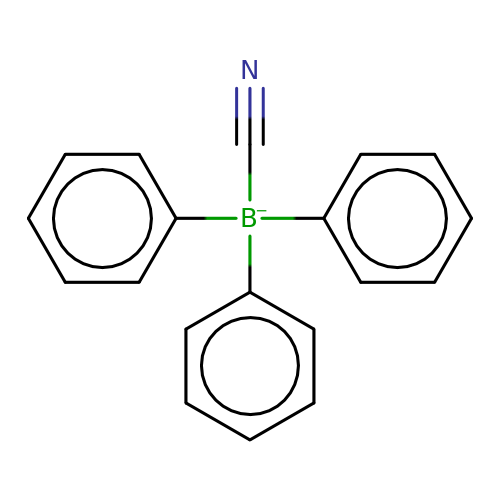 | 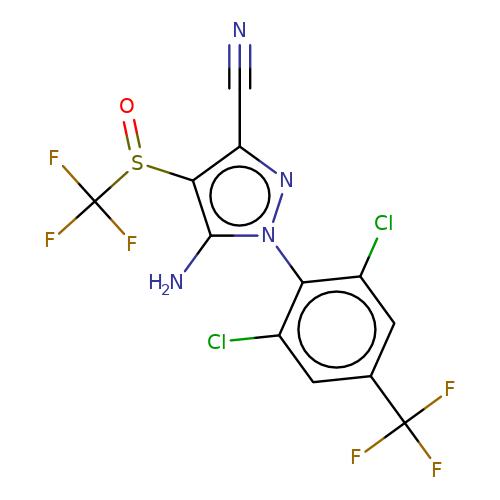 | 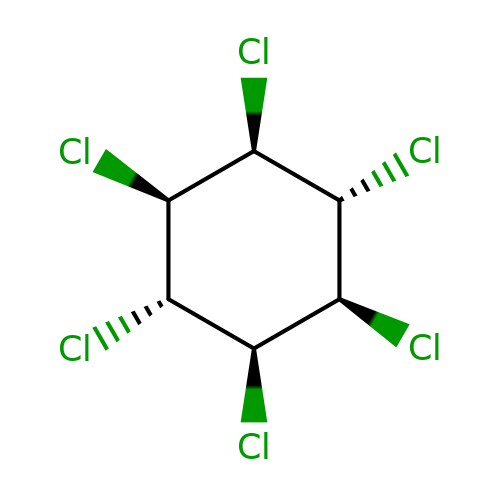 |
| 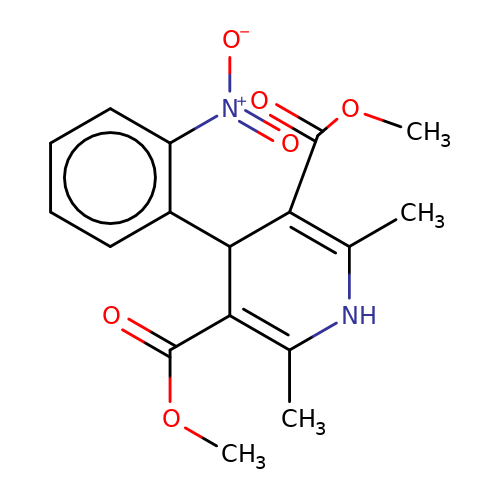 | 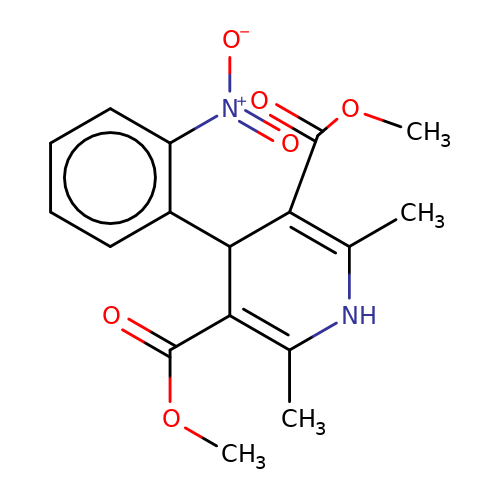 | 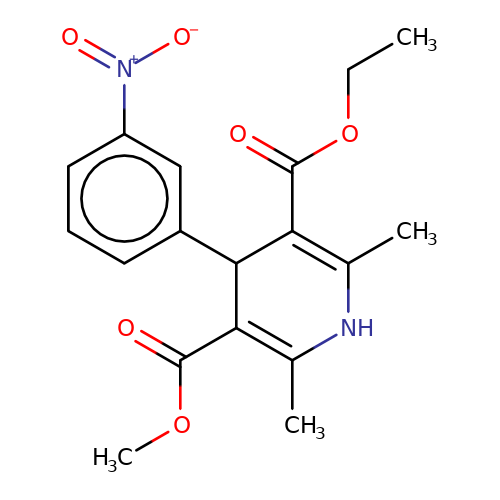 |  |  |  |

**Figure S5.** Chemical structures of GRALL ligands binding within the *ion pore site*. All known ligands binding there are NAMs.

| 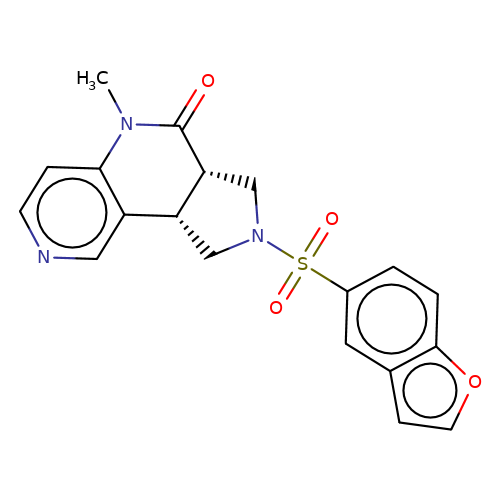 | 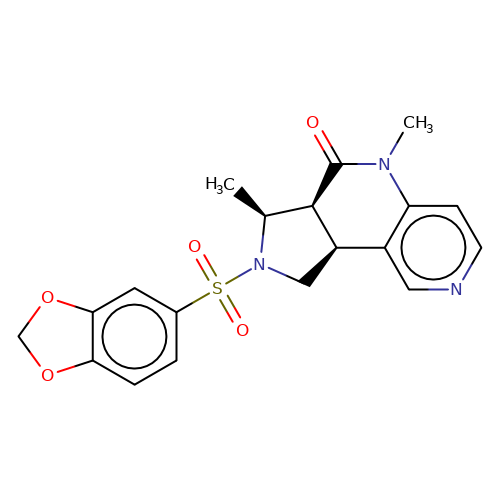 | 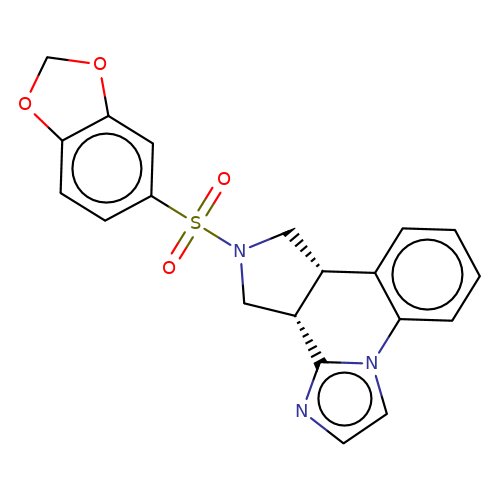 | 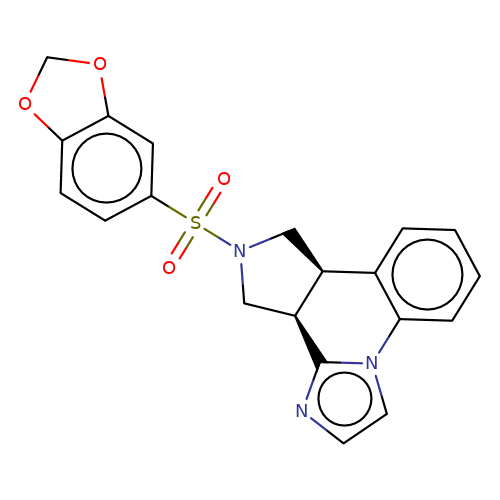 | 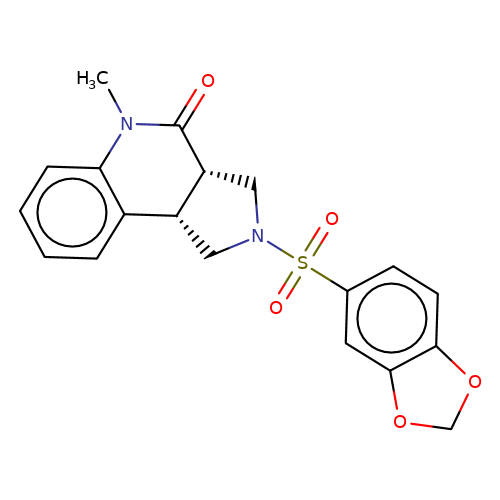 | 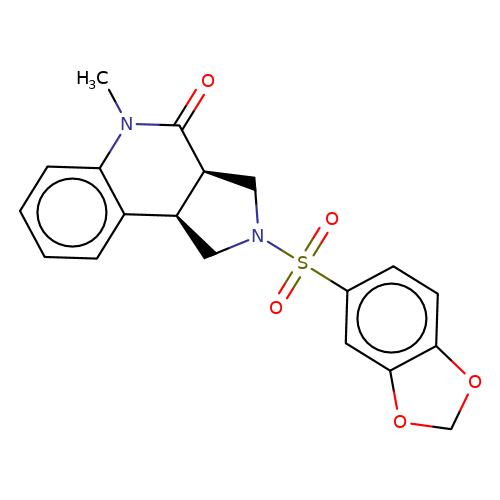 |
| --- | --- | --- | --- | --- | --- |
| 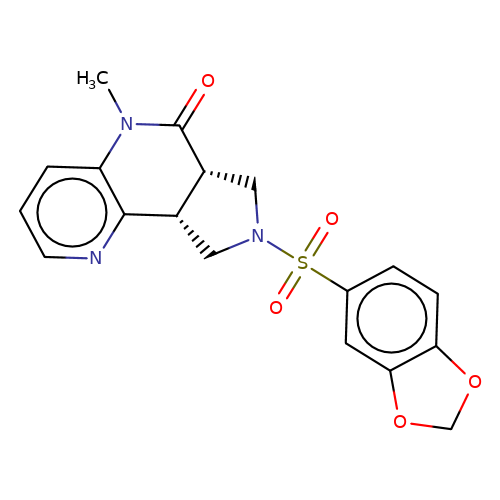 | 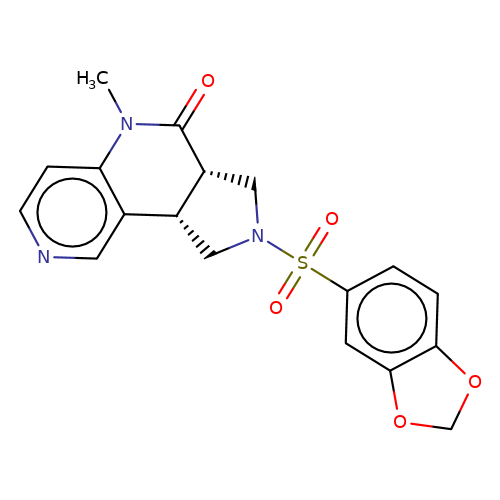 | 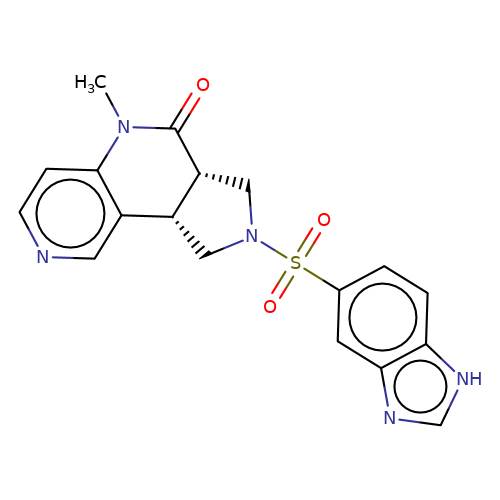 | 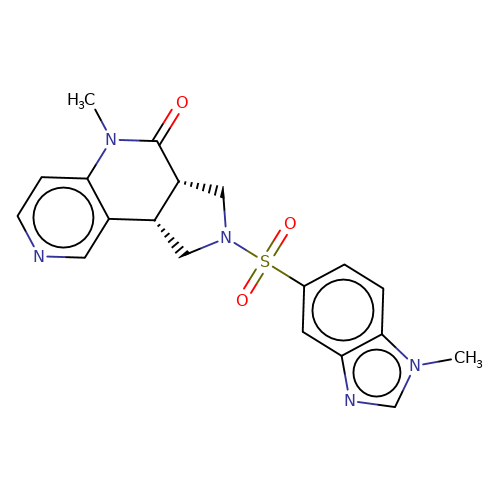 | 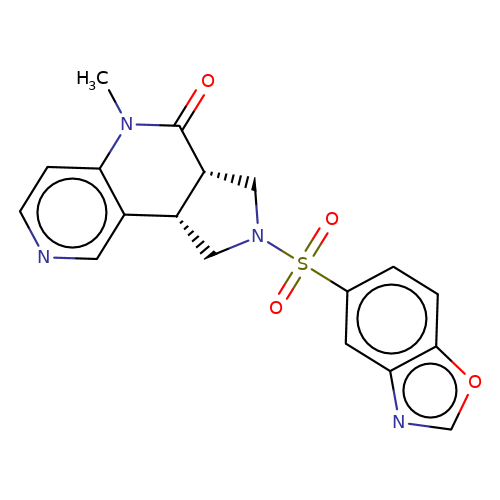 | 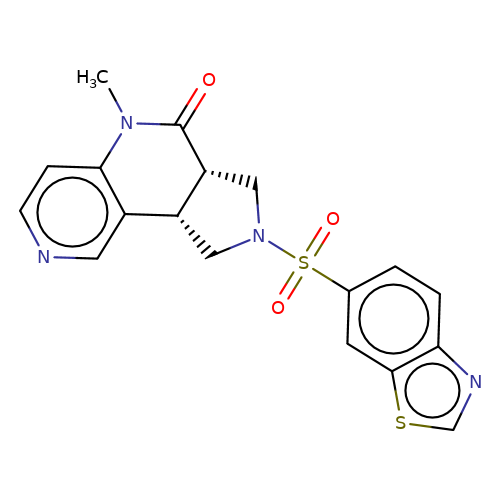 |
| 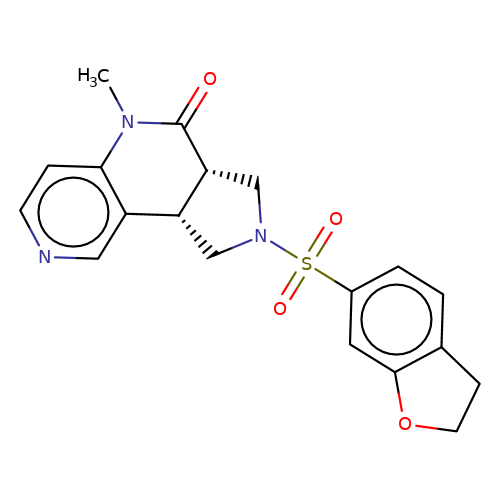 | 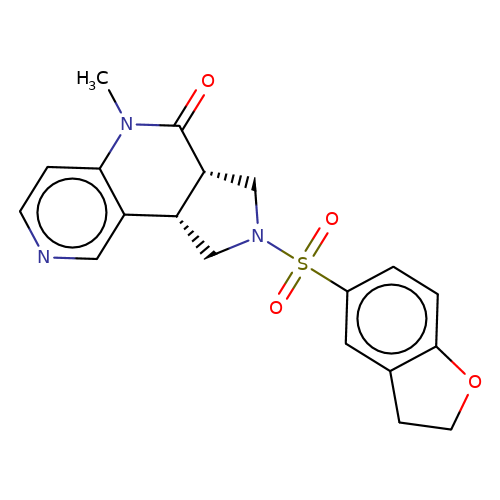 | 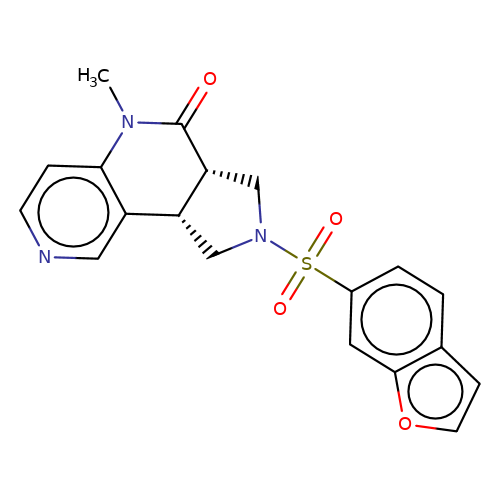 | 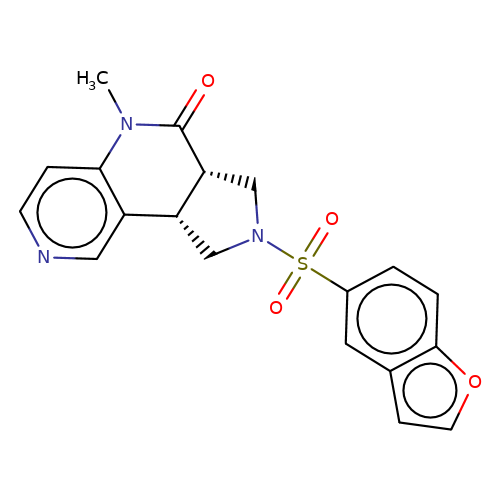 | 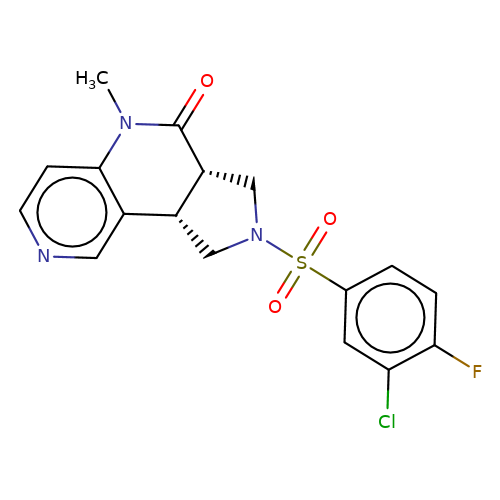 | 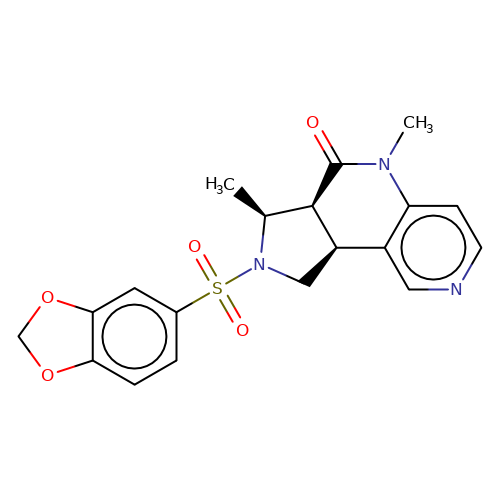 |
| 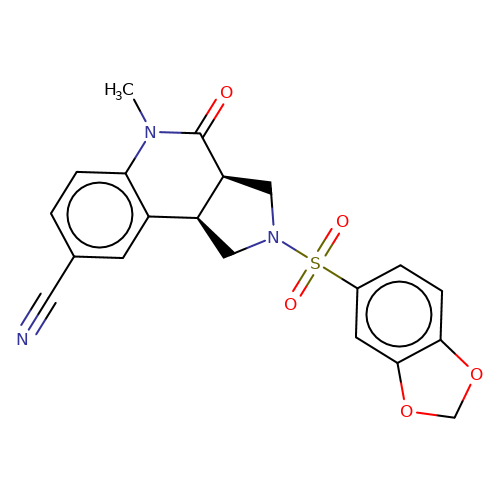 | 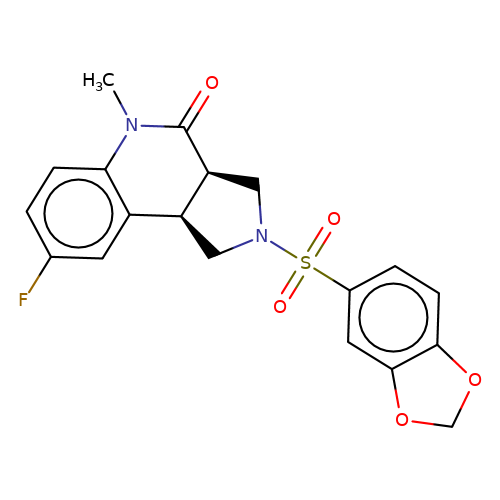 | 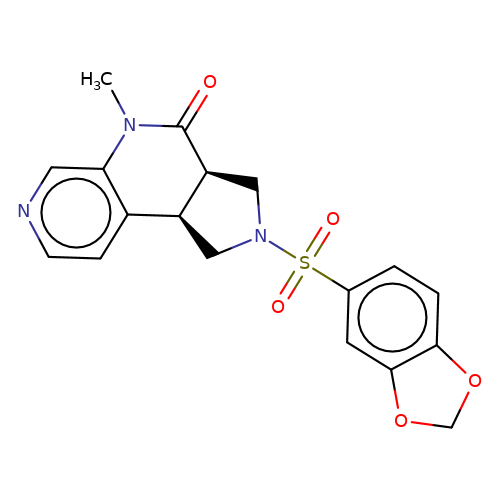 | 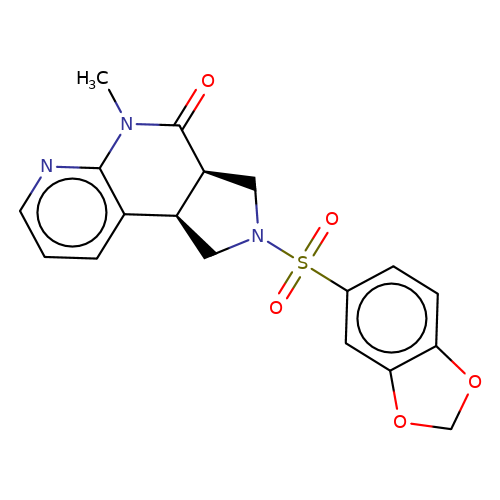 | 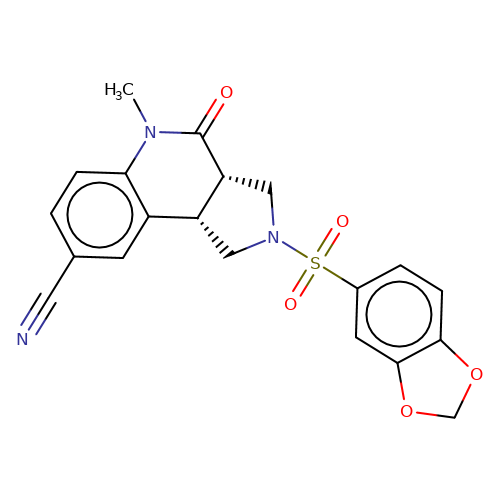 | 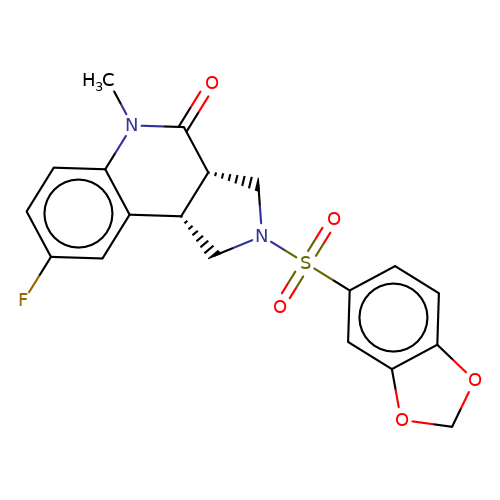 |
| 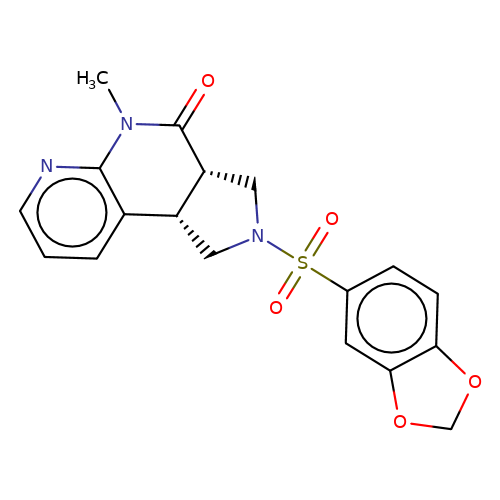 | 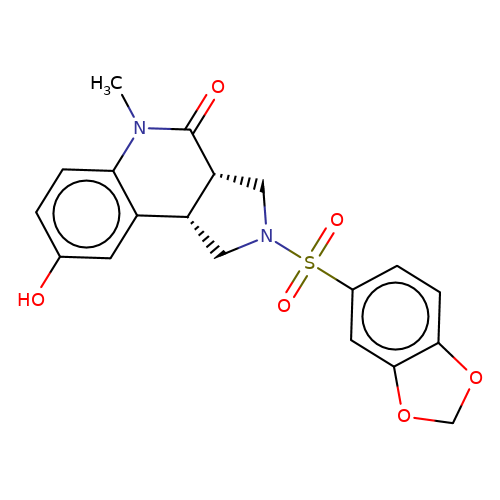 | 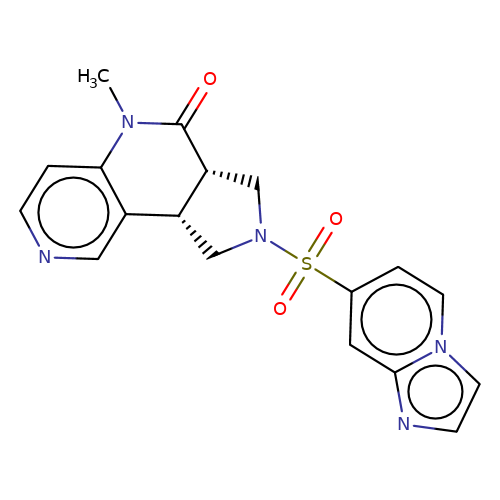 | 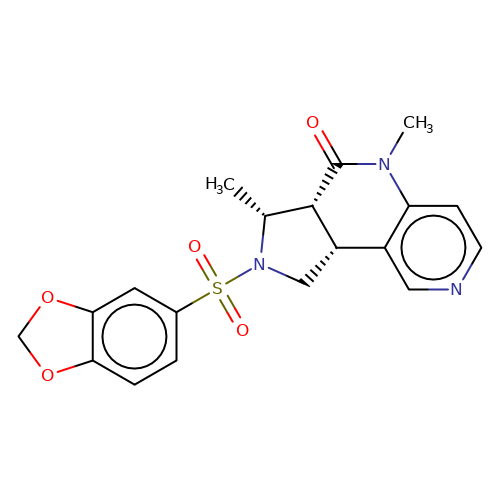 | 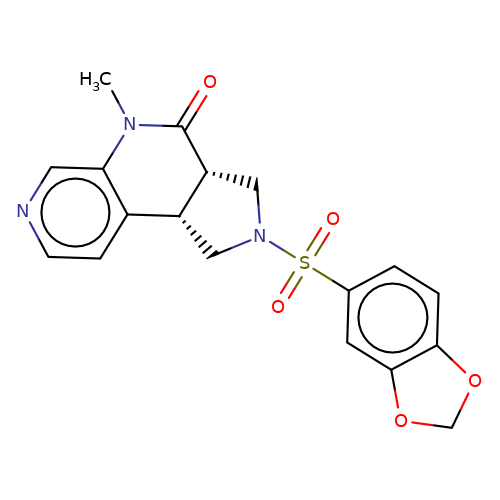 |  |

**Figure S6.** Chemical structures of GRALL ligands binding to the *topECD* *site*. All known ligands binding there are PAMs.

| 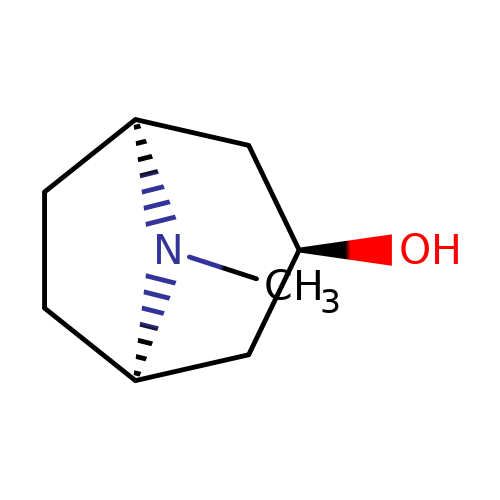 | 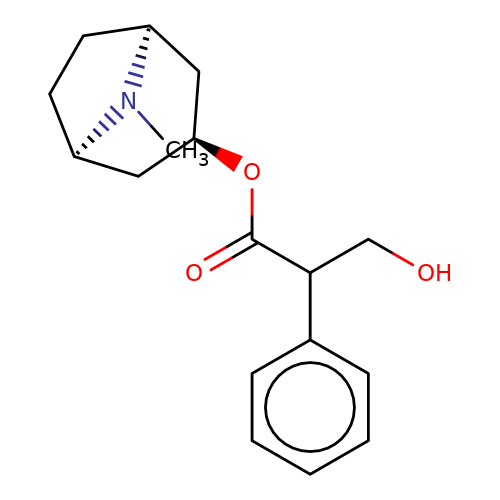 | 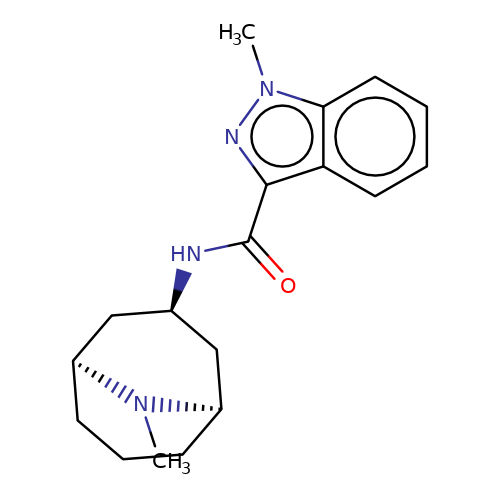 | 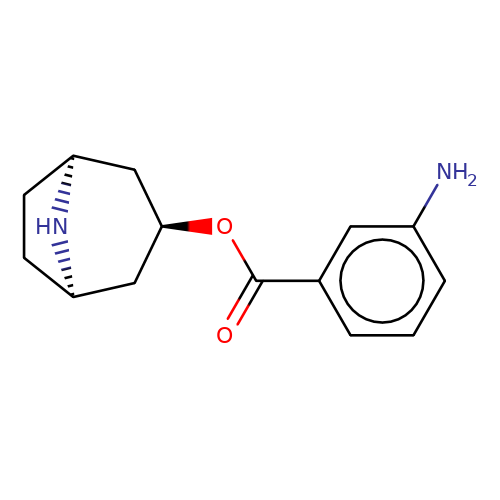 | 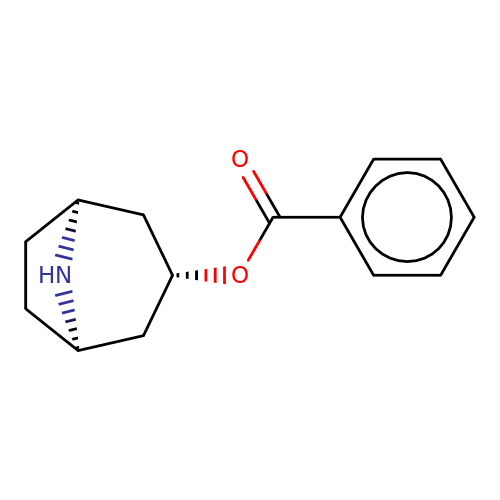 |
| --- | --- | --- | --- | --- |
| 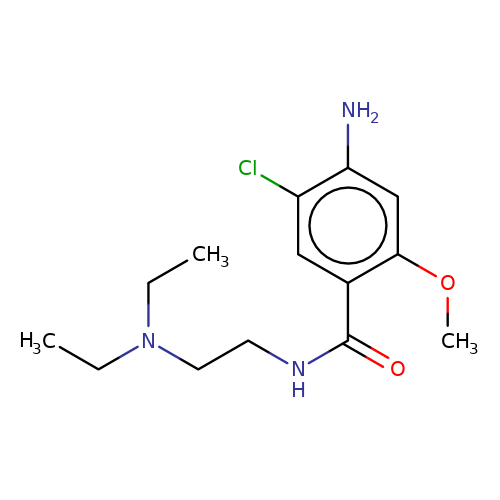 | 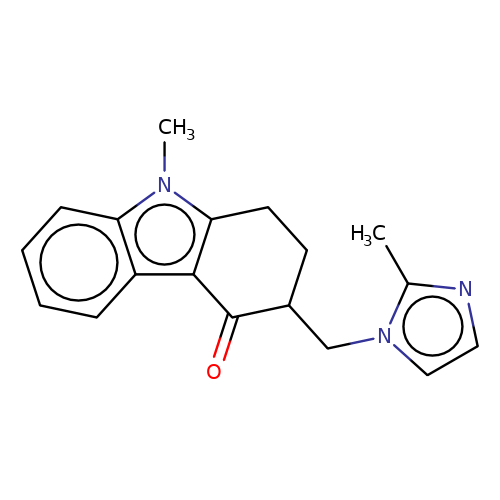 | 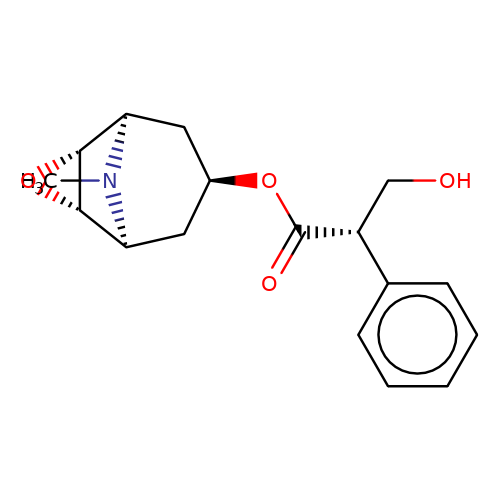 | 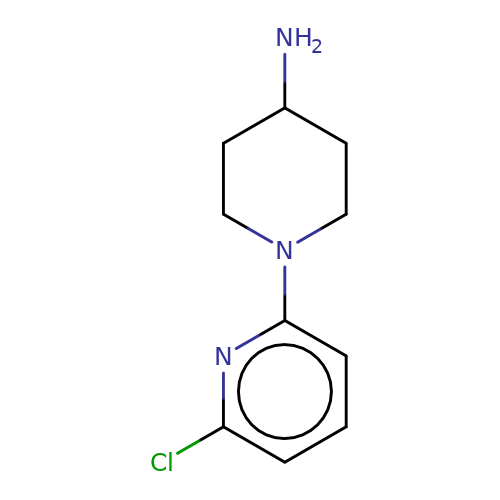 | 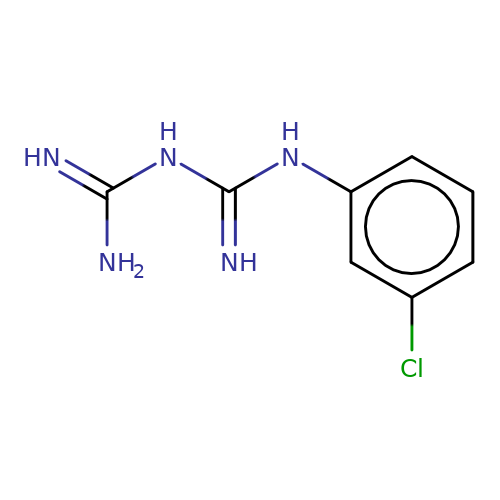 |
| 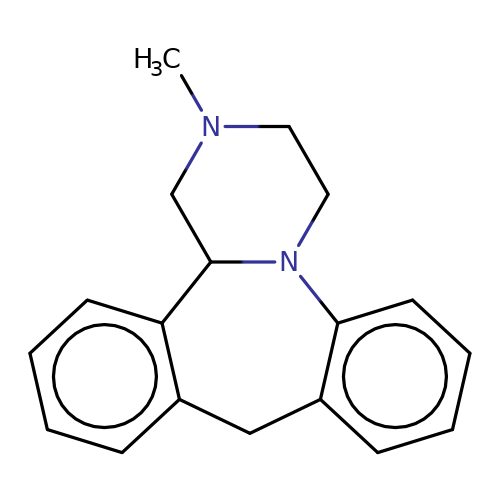 |  |  |  |  |

**Figure S7.** Chemical structures of GRALL ligands binding to the *(low-affinity)-tropeine* *site*. All known ligands binding there are NAMs.

| 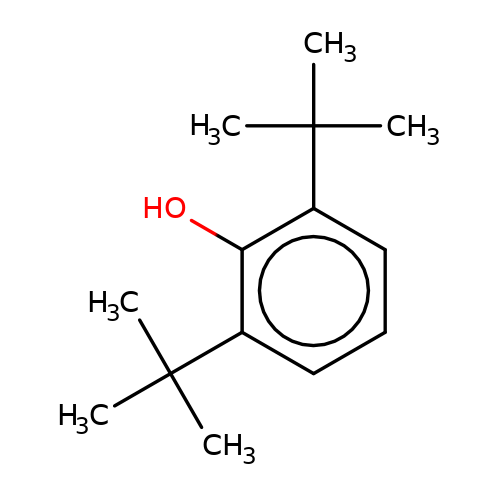 | 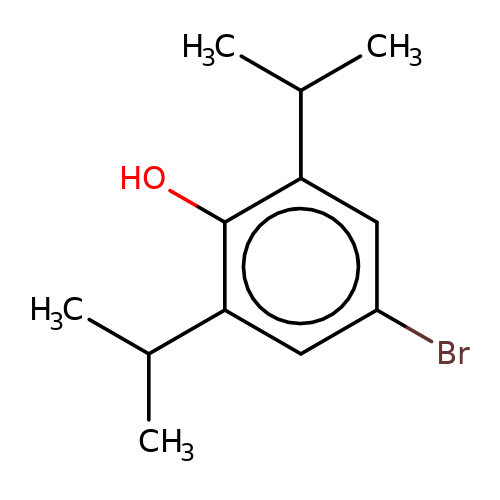 | 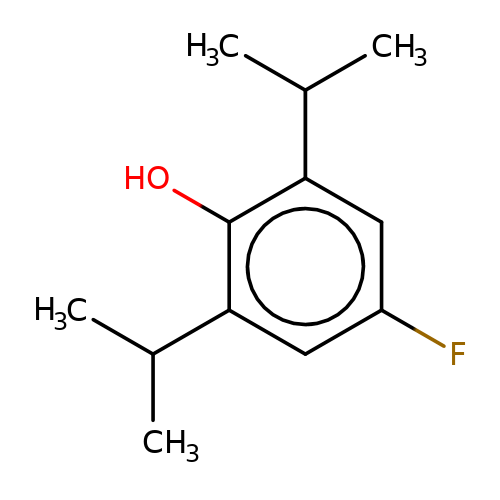 | 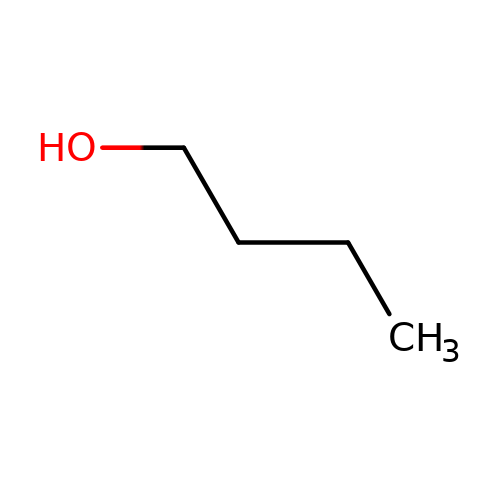 | 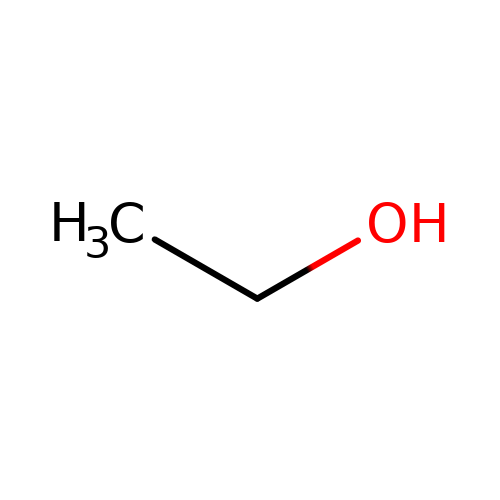 |
| --- | --- | --- | --- | --- |
| 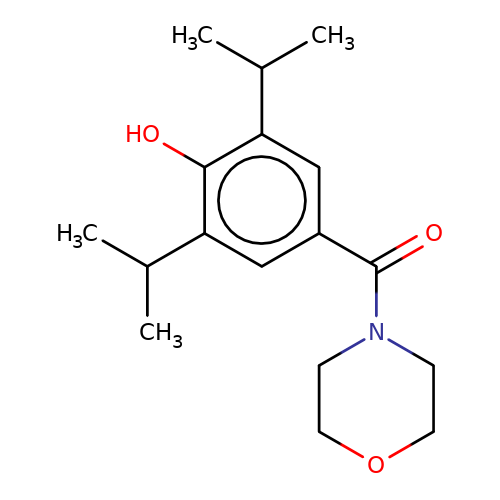 | 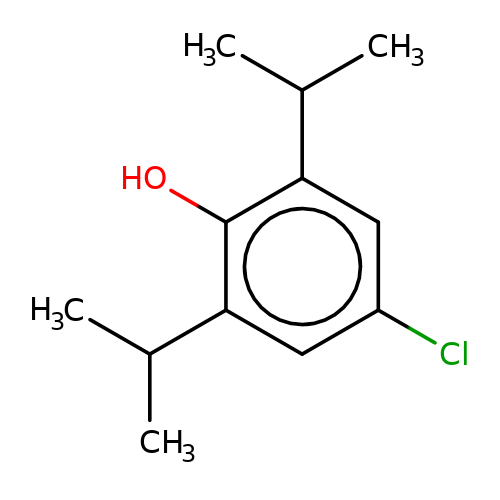 | 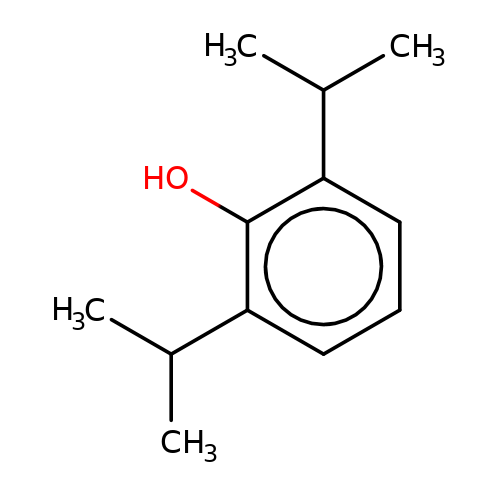 |  |  |

**Figure S8.** Chemical structures of GRALL ligands binding to the *alcohol* *site*. All known ligands binding there are PAMs.

Alvarez, Lautaro D., and Adali Pecci. 2019. “Mapping the Neurosteroid Binding Sites on Glycine Receptors.” *The Journal of Steroid Biochemistry and Molecular Biology* 192 (September): 105388. https://doi.org/10.1016/j.jsbmb.2019.105388.

Fourati, Zaineb, Rebecca J. Howard, Stephanie A. Heusser, Haidai Hu, Reinis R. Ruza, Ludovic Sauguet, Erik Lindahl, and Marc Delarue. 2018. “Structural Basis for a Bimodal Allosteric Mechanism of General Anesthetic Modulation in Pentameric Ligand-Gated Ion Channels.” *Cell Reports* 23 (4): 993–1004. https://doi.org/10.1016/J.CELREP.2018.03.108.

Laverty, Duncan, Philip Thomas, Martin Field, Ole J Andersen, Matthew G Gold, Philip C Biggin, Marc Gielen, and Trevor G Smart. 2017. “Crystal Structures of a GABAA-Receptor Chimera Reveal New Endogenous Neurosteroid-Binding Sites.” *Nature Structural & Molecular Biology* 24 (11): 977–85. https://doi.org/10.1038/nsmb.3477.

Lynagh, T., and B. Laube. 2014. “Opposing Effects of the Anesthetic Propofol at Pentameric Ligand-Gated Ion Channels Mediated by a Common Site.” *Journal of Neuroscience* 34 (6): 2155–59. https://doi.org/10.1523/JNEUROSCI.4307-13.2014.

Maksay, G., B. Laube, and H. Betz. 2001. “Subunit-Specific Modulation of Glycine Receptors by Neurosteroids.” *Neuropharmacology* 41 (3): 369–76. https://doi.org/10.1016/S0028-3908(01)00071-5.

Maksay, Gábor, and Tímea Bíró. 2002. “Dual Cooperative Allosteric Modulation of Binding to Ionotropic Glycine Receptors.” *Neuropharmacology* 43 (7): 1087–98. https://doi.org/10.1016/S0028-3908(02)00213-7.

Maksay, Gábor, Bodo Laube, Rudolf Schemm, Joanna Grudzinska, Malgorzata Drwal, and Heinrich Betz. 2009. “Different Binding Modes of Tropeines Mediating Inhibition and Potentiation of Α1 Glycine Receptors.” *Journal of Neurochemistry* 109 (6): 1725–32. https://doi.org/10.1111/j.1471-4159.2009.06083.x.

Miller, Paul S, Suzanne Scott, Simonas Masiulis, Luigi De Colibus, Els Pardon, Jan Steyaert, and A Radu Aricescu. 2017. “Structural Basis for GABAA Receptor Potentiation by Neurosteroids.” *Nature Structural & Molecular Biology* 24 (11): 986–92. https://doi.org/10.1038/nsmb.3484.

Weir, C.J., A.T.Y. Ling, D Belelli, J.A.W. Wildsmith, J.A. Peters, and J.J. Lambert. 2004. “The Interaction of Anaesthetic Steroids with Recombinant Glycine and GABA A Receptors †.” *British Journal of Anaesthesia* 92 (5): 704–11. https://doi.org/10.1093/bja/aeh125.

Yang, Zhe, Agnieszka Ney, Brett A. Cromer, Hooi-Ling Ng, Michael W. Parker, and Joseph W. Lynch. 2007. “Tropisetron Modulation of the Glycine Receptor: Femtomolar Potentiation and a Molecular Determinant of Inhibition.” *Journal of Neurochemistry* 100 (3): 758–69. https://doi.org/10.1111/j.1471-4159.2006.04242.x.
